# A reformulation of pLSA for uncertainty estimation and hypothesis testing in bio-imaging

**DOI:** 10.1093/bioinformatics/btaa270

**Published:** 2020-04-23

**Authors:** P D Tar, N A Thacker, S Deepaisarn, J P B O’Connor, A W McMahon

**Affiliations:** b1 Division of Informatics, Imaging and Data Sciences; b2 Division of Cancer Sciences, The University of Manchester, M13 9PG Manchester, UK

## Abstract

**Motivation:**

Probabilistic latent semantic analysis (pLSA) is commonly applied to describe mass spectra (MS) images. However, the method does not provide certain outputs necessary for the quantitative scientific interpretation of data. In particular, it lacks assessment of statistical uncertainty and the ability to perform hypothesis testing. We show how linear Poisson modelling advances pLSA, giving covariances on model parameters and supporting $\chi^{2}$ testing for the presence/absence of MS signal components. As an example, this is useful for the identification of pathology in MALDI biological samples. We also show potential wider applicability, beyond MS, using magnetic resonance imaging (MRI) data from colorectal xenograft models.

**Results:**

Simulations and MALDI spectra of a stroke-damaged rat brain show MS signals from pathological tissue can be quantified. MRI diffusion data of control and radiotherapy-treated tumours further show high sensitivity hypothesis testing for treatment effects. Successful $\chi^{2}$ and degrees-of-freedom are computed, allowing null-hypothesis thresholding at high levels of confidence.

**Availability and implementation:**

Open-source image analysis software available from TINA Vision, www.tina-vision.net.

**Supplementary information:**

Supplementary data are available at *Bioinformatics* online.

## 1 Introduction

When reporting scientific discoveries, it is conventional to quantify the significance of results via hypothesis testing. A null-hypothesis is formed assuming there is no effect beyond measurement noise. If signal deviations exceed a probability threshold then this is considered evidence of a possible effect. A medical intervention will typically be considered potentially effective only if it reaches a *P*-value below 0.05 (Fisher, 1925)—a result that requires caution as it can be caused by noise 1 in 20 times. Alternatives to hypothesis testing include dichotomy testing, where one hypothesis is tested against another [e.g. using Likelihood ratios (McGee, 2002)], or Bayesian selection (Raftery, 1995) where multiple hypotheses are simultaneously tested. This work develops a sensitive hypothesis testing method for bio-images, particularly suited to Poisson mass spectra (MS) data (i.e. ion counts), but also applicable to other source of Poisson data, such as binned counts of voxels sampled from medical imagery [e.g. magnetic resonance imaging (MRI) data].

An honest significance test will produce a uniform distribution of *P*-values when observations are drawn from data consistent with the null-hypothesis (Welch and Peers, 1963). This honesty can be lost if models are inappropriately selected or data assumptions are violated. Achieving this uniformity is a key measure of success for our proposed hypothesis testing method. We argue that rigorous quality control is necessary before results can be considered trustworthy and that success on one dataset does not guarantee future success for all similar data. Tools, such as Bland-Altman analysis (Bland and Altman, 1986), must be used to check noise characteristics as a function of observed value, e.g. that Poisson noise grows with the square-root of the observed measurement. Pull distributions (Demortier and Lyons, 2002) can be used to check that errors on parameters match those predicted by theory. Monte Carlo simulation is a further tool for testing software under controlled conditions. By applying such testing, it is possible to determine when results can be trusted.

A common approach to hypothesis testing is to inspect mode fit residuals. This can be achieved using $\chi^{2}$ statistics which requires: Gaussian residuals (an assumption); scaling of residuals to unit variance (requiring knowledge of error size); and knowledge of the number of degrees-of-freedom (d.f.) remaining after parameter estimation (which can also correct for residual correlations). Such knowledge may be readily available for simple regressions, but can be harder to determine in complex data and machine-learning systems. The modelling of MS images of biological specimens is a case in point, where dimensionality changes from spectrum-to-spectrum (via multiple changing tissues) and ion counts can vary giving different levels of uncertainty (via changing signal-to-noise). Whilst methods exist for assessing processes in diseased and injured tissue for MS (e.g. Henderson *et al.*, 2018), additional work is needed for $\chi^{2}$ testing, e.g. for *P*-values on pathology.

Linear decompositions, such as PCA and ICA (Gut *et al.*, 2015), extract correlations between mass peaks. Under these methods, peaks that covary are represented as elements of orthogonal (PCA) or non-orthogonal (ICA) vectors. It is reported that ICA methods are promising for use with MS (Nicolaou *et al.*, 2011). However, PCA and ICA are usually based upon Gaussian noise assumptions and can include both positive and non-physical negative loadings. MS, in contrast, are positive-only and, depending upon the type of spectra, can be dominated by Poisson sampling noise (Harn *et al.*, 2015; Piehowski *et al.*, 2009) in regions where peaks are well populated. Linear models also have an advantage over machine-learning classification labelling systems. At the resolution of MS images, multiple tissue types can exist within a single spectrum, thus a linear composition model is a more sensible description than a set of class labels. These characteristics have led to an industry standard approach for such MS modelling called probabilistic latent semantic analysis (pLSA), as found in Bruker’s SCiLs lab software. Our similar modelling method, linear Poisson modelling (LPM), has previously been used to make measurements of tissue mixtures from MALDI MS (Deepaisarn *et al.*, 2018).

Most approaches, including pLSA, do not output information needed for $\chi^{2}$ testing as a standard part of their algorithm. Some work has been undertaken to determine the error terms for some of these methods (Tang and Nehorai, 2011) to give parameter covariances on model weights via application of the minimum variance bound (MVB), but fall short of hypothesis testing. In state-of-the-art machine learning, aleatoric (observational) and epistemic (encoded model) uncertainties have been estimated for Bayesian deep learning (Kendall and Gal, 2017), but these do not directly equate to traditional statistical and systematic errors, nor are d.f. calculated making it inappropriate for $\chi^{2}$ testing. The novelty of this work is demonstrating that our (LPM) (Tar *et al.*, 2015) method is appropriate, and can be extended to provide all the necessary outputs for *P*-values to be estimated from $\chi^{2}$ values—primarily for MS, but also for different imaging modalities, such as MRI. However, successful application is dependent upon results passing quality control criteria, as is achieved with our example data.

## 2 Materials and methods

**Fig. 1.**
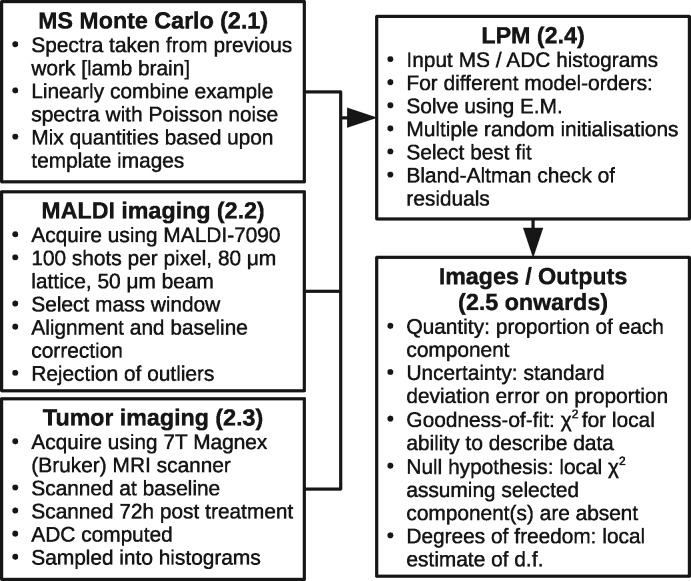
Outline of work-flow. Numbers indicate related method sub-sections

To validate spectrum-by-spectrum $\chi^{2}$ testing on MALDI images and simulated MS data, the followed steps are taken (outline in Fig. 1):

- Preparation and pre-processing of MS is performed to approximate statistical properties needed for LPM. Simulated MS are generated with independent Poisson noise and known ground-truth for validation.
- An appropriate model-order is needed, fitted sufficiently close to a global solution to give well-behaved residuals. Quality is checked using a global $\chi^{2}$ (all data) and also Bland–Atman analysis.
- Per-spectrum model weight error covariances must be estimated and validated. Validation can be performed using known ground-truth and pull distributions.
- Per-spectrum error predictions are used to estimate the effect of model fitting on residuals, allowing an effective number of d.f. to be calculated for individual spectra.
- A model component is selected to play the role of ‘null’ component. This forms a null-hypothesis that the component is not required to describe the data. A $\chi^{2}$ is computed for each fitted spectrum with and without the null component in order to compute *P*-values.
- Regions of data believed to be consistent with the null-hypothesis are tested to check if the *P*-value distribution is uniform, as it should be when driven only by noise. Regions that are strongly not consistent with the null-hypothesis are revealed by applying a *P*-value threshold.

MALDI imaging has been widely applied to study rat brain models. Current literature linking lipids with this well-known anatomy makes this target a useful demonstrator. We select a rat brain image post-ischaemic stroke [data originating from Henderson *et al.* (2018)], as despite having no exact quantitative ground-truth, the location of stroke damaged is known. A component that highly correlates with this region is used as the null-hypothesis component. The hypothesis test will therefore act as a test for pathology. The image contains over 25 000 spectra covering multiple tissue types (e.g. white matter, grey matter, fat, CSF spaces etc.).

The modelling of more general histogram data, in the form of sampled data distributions from MRI scans, is considered as a second dataset, requiring fewer pre-processing steps. We build LPM descriptions of diffusion-weighted MRI data acquired in colorectal xenograft tumours (LoVo and HCT116), where the apparent diffusion co-efficient (ADC—a measure of water mobility within tissues) was calculated for each image voxel. Here, ADC distributions, one per tumour, are sampled into histograms, where each histogram plays a similar role to a mass spectrum. In this case, the null-hypotheses are that there are no measurable treatment effects. A control model is fitted to untreated and also radiotherapy-treated distributions, to demonstrate how the resulting $\chi^{2}$ can be used to differentiate between both groups.

### 2.1 Monte Carlo MS images

**Fig. 2.**
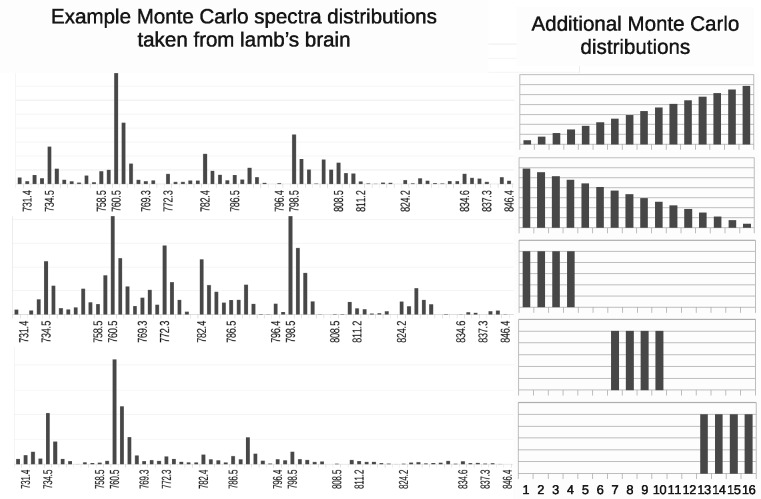
Spectra used as source components to build simulated MS images. These include real MALDI spectra from lamb’s brain tissue and also simple distributions to test a wide range of overlapping bins (via the ramps) and unique bins (top-hats)

**Fig. 3.**
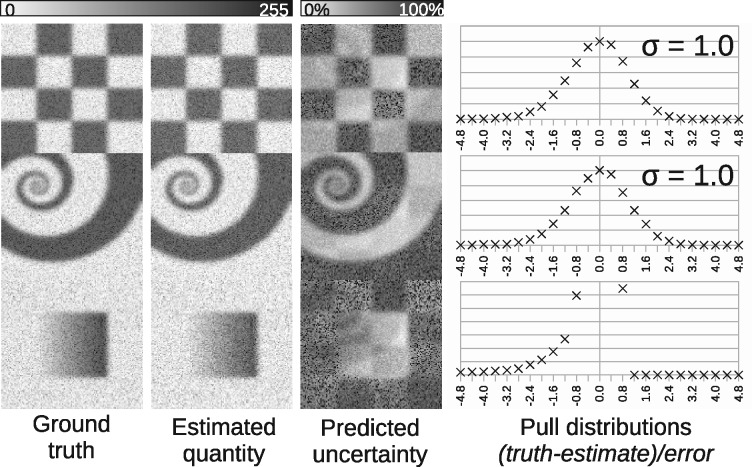
Results of modelling synthetic MS images. From left to right: ground-truth mixing quantities; LPM quantity estimates; LPM predicted uncertainties; pull distributions showing that deviations from ground-truth are consistent with predicted levels of uncertainty

Simulated images (128×128 pixels) were generated by linearly weighting example spectra from previously published work (Deepaisarn *et al.*, 2018, white and grey matter from lamb brain), and also simple ramps and top-hat distributions (Fig. 2). Ground-truth quantities were set using images of overlapping geometric shapes to simulate mixed tissues (Fig. 3). A designated ‘pathology’ component covers a rectangle with a smooth gradient to simulate pixels containing from 0 to ∼33% pathology. To simulate a range of sample sizes, the expected ion quantities per-pixel are draw from a Gaussian distribution with the mean set to the typical levels of signal found within the real rat brain image. A Poisson random number generator is then used to draw individual ion counts following these selected means. In all cases, artefacts that are removed through the pre-processing describe below (baseline, shifting, etc.) are not simulated, thus Monte Carlo data were suitable for direct LPM analysis.

### 2.2 MS acquisition and pre-processing

The MS data used were taken from Henderson *et al.* (2018). A ligature of the left middle cerebral artery trunk and common carotid artery induced an ischaemic stroke in a Wistar rat. After 3 months recovery, the brain was sectioned into 12 *μ*m coronal slices. Sections were washed and a matrix of 2,5-dihydroxybenzoic acid applied. Imaging was performed using a MALDI-7090 mass spectrometer by Shimadzu, Manchester, UK. MS were acquired with a spatial resolution of 80 and 50 *μ*m beam diameter using positive ion reflectron mode. A total of 100 shots were accumulated per pixel. A mass window of 690–890 m/z was selected to coincide with known lipids and to exclude low mass matrix-related contamination. A visual inspection confirmed that there were no atypical peak distributions in comparison to adjacent pixels that might indicate poor acquisitions or outliers.

Baseline correction and relative mass calibration is required to mitigate against non-Poisson effects. Software developed in Thacker *et al.* (2018) was used to perform a Fourier domain peak alignment, estimate and remove a smooth background and identify and integrate bins with significant peaks. Peak identification is performed on the average total spectrum from all spectra after alignment. Peaks span multiple bins in the original raw MS. The central m/z value within the range is tentatively assigned to the integrated pre-processed peaks, accurate to ∼±0.5 m/z. The Poisson behaviour of resulting mass histograms was previously confirmed via Bland–Altman analysis (Bland and Altman, 1986). The resulting pre-processed image data contained 25 842 spectra, each containing 67 peaks.

### 2.3 MRI acquisition and pre-processing

The ADC distributions used here were taken from Tar *et al.* (2018), where full details can be found. Additional information is also provided in the Supplementary Material. Mice bearing colorectal xenografts (LoVo and HCT116) were imaged at baseline and also 72 h post-treatment. *n*=8 (LoVo) and *n*=13 (HCT116) control group tumours were untreated and 10 (LoVo) and 15 (HCT116) were treated with radiotherapy. ADC values were computed for each imaged volume at both time points, and sampled into 2D histograms of ACD versus time.

### 2.4 Modelling

LPM has much in common with pLSA (Hofmann, 1999) and non-negative matrix factorization (NNMF) (Lee and Seung, 1999). These impose a realistic non-negative interpretation on spectra, make a multi-nomial noise assumption that tends towards Poisson statistics with large numbers of mass bins, and produce generative models (Hanselmann *et al.*, 2008). The connection between the methods can be seen in their linear manifolds and cost-functions. LPM can be viewed as a reformulation of pLSA and NNMF with a Poisson noise assumption and different parameter normalizations. However, LPM includes: the MAX SEP algorithm to reduce linear degeneracies; an error theory for predicting covariances; and introduced here, the ability to perform local hypothesis testing.

Semantic analyses originated in the modelling of documents, where a corpus of text is decomposed into categories of writing styles. Under pLSA, the frequency of word occurrences can be described using a linear combination of probability distributions:

(1) $$M_{is}^{\mathrm{pLSA}}=P(i,s)=\sum_{k}P(k)P(s|k)P(i|k),$$

where *P*(*i*, *s*) is the probability of drawing an instance of word *i* in a source document *s*; *P*(*k*) is the probability of drawing a source document of type *k* from the corpus; P(s|k) is the probability of drawing source document *s* given that it is of type *k*; and P(i|k) is the probability of drawing an instance of word *i* given that it is from a document of type *k*. In contrast, NMF uses linear models based upon non-normalized positive-only values rather than probability distributions:

(2) $$H_{is}\approx M_{is}^{\mathrm{NNMF}}=\sum_{k}W_{ks}F_{ik},$$

where $H_{is}$ is the frequency (e.g. histogram) of words *i* in document *s* that is approximated by the model; and $F_{ik}$ is an un-normalized distribution of words *i* found within documents of type *k*; and $W_{ks}$ are the weights required to describe the histogram. **F** and **W** are therefore interpretable as non-negative matrices. It has been shown that NNMF and pLSA optimize equivalent cost-functions and therefore produce equivalent linear descriptions of data (Ding *et al.*, 2008), albeit with different parameter normalizations and different optimization algorithms.

Both of these methods can be used to describe MS images by making *k* a type of tissue, *i* (or *s*) an ion in a spectrum and *s* (or *i*) a spectrum at a given pixel. It is then up to the user (or software developer) to keep track of normalizations and their physical meanings for the interpretation of tissue proportions at different pixels. LPM uses a more intuitive model that describes spectra using a mixture of probability distributions and un-normalized quantities:

(3) $$H_{is}\approx M_{is}^{\mathrm{LPM}}=\sum_{k}Q_{ks}P(i|k),$$

where P(i|k) is the conditional probability distribution of ions *i* within tissue type *k*; and $Q_{ks}$ is the quantity of signal from tissue *k* present within spectrum *s*. An advantage under this model is that a spectrum associated with a tissue is neatly described using a simple probability mass function (PMF) and the amount of signal (e.g. in millivolts) is an absolute quantity.

LPM is equivalent to pLSA (and therefore NNMF also), both in its linear model and cost-function. The joint probability, *P*(*i*, *s*), given by Equation (1), describes the distribution of the frequency data, $H_{is}$, given by Equation (3), normalized to the total quantity, $T=\sum_{k}\sum_{s}Q_{ks}$, therefore:

(4) $$\begin{array}{c} TM_{is}^{\mathrm{pLSA}}=T\sum_{k}P(k)P(s|k)P(i|k)\\ =\sum_{k}TP(k,s)P(i|k)=\sum_{k}Q_{ks}P(i|k)=M_{is}^{\mathrm{LPM}}, \end{array}$$

so pLSA and LPM share a linear model to within a scaling factor. Turning to the cost-function, pLSA maximizes the log-Likelihood:

(5) $$\log L=\sum_{i}\sum_{s}H_{is}\;\text{log}(M_{is}^{\mathrm{pLSA}}),$$

whereas LPM maximizes the *extended* log-likelihood:

(6) $$\log L=\left[\sum_{i}\sum_{s}H_{is}\;\text{log}(TM_{is}^{\mathrm{pLSA}})\right]-T,$$

where the former is for a fixed total quantity of events (e.g. counted ions) and the latter is compatible with a Poisson model where the total quantity of events has an expectation of *T*. It has been shown by Barlow (1990) that optimizing equivalent parameters using maximum likelihood or extended maximum likelihood produces the same statistical estimates, but with different MVB. This equivalence means that the three approaches describe the same manifold, assuming a good optimum is found. LPM fits a linear combination of arbitrary PMFs to a cohort of histograms using expectation–maximization (EM) (Tar *et al.*, 2018), similarly to how pLSA uses EM. Each algorithm is iterative and numerical in nature so all suffer the same indeterministic solutions. This can be mitigated by using multiple random restarts and the use of a goodness-of-fit for finding the best solutions out of many.

If a finite amount of each tissues’ chemicals are always present, it is more difficult to determine the location of zero loadings needed to identify each tissue uniquely. To mitigate this problem, PMFs are post-processed using an algorithm, we call MAX SEP (Deepaisarn *et al.*, 2018). MAX SEP attempts to subtract some quantity, *α*, of each component from every other, as far as possible, without generating any negative probabilities. This makes each component more compact by increasing the number of zero valued bins, whilst also maximizing the positive-only volume of the models’ linear sub-space:

(7) P′(i|k)=P(i|k)−αP(i|l),

(8) $$\arg_{\alpha }\max P\prime (i|k):=\{\alpha |\forall i:P(i|k)-\alpha P(i|l)\geq 0\}.$$

A renormalization step, followed by further application of the EM loop can converge upon the simplified components. MAX SEP is expected to be most useful for separating sub-spectra when multiple tissues exist within each spectrum. Satisfying the ‘simple structure’ criteria, via MAX SEP, should produce more repeatable and physically meaningful components.

The number of components, *N* (the model-order), required to fully describe a histogram cohort is determined using a model selection process. Multiple models are constructed with increasing numbers of components until a satisfactory goodness-of-fit is achieved. For each value of *N*, a total of five models are constructed from different random starting parameters so that the best fitting solution from a pool of possible local minima is selected. The associated LPM error theory allows for the prediction of model-data residuals, permitting absolute goodness-of-fits to be computed in the form of a $\chi^{2}$ per d.f.:

(9) $$\chi_{D}^{2}=\frac{1}{D}\sum_{i}\sum_{s}\frac{{\left(\sqrt{H_{is}}-\sqrt{M_{is}}\right)}^{2}}{\sigma_{is}^{2}},$$

where *D* is the d.f. and $\sigma_{is}^{2}$ is the residual variance associated with m/z range *i* in spectrum *s*. The square-roots transform the Poisson distributed histogram frequencies into Gaussian-like variables to improve this figure of merit’s approximation to ideal $\chi^{2}$ statistics, following Anscombe (1948). Note that, this is a global $\chi^{2}$ describing the goodness-of-fit of the LPM for an entire image (i.e. over all pixels and mass bins). The d.f., *D*, is therefore equal to the total number of populated MS bins within the entire image, minus the total number of estimated parameters (i.e. all quantities and PMF elements) for the entire image. The per-spectrum d.f. is dependent upon local pixel dimensionality, as described later.

A curve of Equation (9) against model-order *N* will plateau when the optimal value of *N* is achieved. As bins record voltages rather than direct counts, there will be a scaling factor between histogram bins and Poisson events. The model selection plateau is therefore not guaranteed to reach unity, but the square-root of the plateau value should match the voltage step-change associated with a single count. Bland–Altman analysis can corroborate the scaling factor, thus confirming the expected plateau value. Multiple potential model-orders can be selected close to the lowest $\chi^{2}$, followed by further quality control testing.

### 2.5 Error covariances

The stability of each quantity, $Q_{ks}$, can be estimated using the MVB applied to the LPM likelihood function. This gives an inverse covariance for errors on quantities *a* and *b* in spectrum *s* as:

(10) $$C_{s}^{-1}\approx \frac{-\partial^{2}\;\text{log}\;L}{\partial Q_{as}\partial Q_{bs}}.$$

Inverting then taking the square-root of diagonal terms thus gives the ±1 SD error bar for any spectrum and quantity.

### 2.6 Local $\chi^{2}$ and d.f. estimation

A $\chi^{2}$ for an individual spectrum, *s*, is given by

(11) $$\chi^{2}=\sum_{i}\frac{{\left(\sqrt{H_{is}}-\sqrt{M_{is}}\right)}^{2}}{\sigma_{is}^{2}}.$$

Testing the hypothesis that a quantity is consistent with zero is to say that any finite quantity estimated is caused only by noise. This can be observed in the model residuals by omitting a component of interest from the LPM (assuming it is not needed as the null-hypothesis) and computing a $\chi^{2}$. A residual for a bin *i* is given by

(12) $$\delta_{i}=\sqrt{H_{i}\pm e_{H}}-\sqrt{M_{i}\pm e_{M}},$$

where *H* is the histogram and *M* is the LPM. The histogram has a sampling error, *e_H_*, driven by the Poisson counting process. The model also has an error that may include a contribution from sampling errors during training and in the estimation of quantities. This model is given by

(13) $$M_{i}=\sum_{k}\;[P(i|k)\pm e_{P}][Q(i)\pm e_{Q}],$$

where *e_p_* and *e_Q_* are training and estimation errors, respectively. A $\chi^{2}$ can then be computed for a single histogram using *n* non-zero bins

(14) $$\chi^{2}=\sum_{i=1}^{n}\frac{\delta_{i}^{2}}{\frac{1}{4}+\sigma_{M_{i}}^{2}},$$

where the $\frac{1}{4}$ variance is the fixed variance after the square-root transform and $\sigma_{M}^{2}$ is the variance from model errors. As explained in Section 2.4, the number of d.f. within an individual histogram is not easy to state exactly, as it is a function of the local dimensionality of the data and how well-populated it is. On average, the reduction in variance due to model parameters is $\sigma_{i_{\mathrm{dfc}}}^{2}$, so that

$$<\delta_{i}^{2}>=\frac{1}{4}+\sigma_{M_{i}}^{2}-\sigma_{i_{\mathrm{dfc}}}^{2}.$$

The average $\chi^{2}$ we expect to obtain can be interpreted as the effective number of d.f. (*n*_eff_). An estimate of this quantity can therefore be made for *n* populated bins using

(15) $$n_{\mathrm{eff}}=\sum_{i=1}^{n}\frac{\frac{1}{4}+\sigma_{M_{i}}^{2}-\sigma_{i_{\mathrm{dfc}}}^{2}}{\frac{1}{4}+\sigma_{M_{i}}^{2}}=\sum_{i=1}^{n}1-\frac{\sigma_{i_{\mathrm{dfc}}}^{2}}{\frac{1}{4}+\sigma_{i_{X}}^{2}},$$

where $\sigma_{i_{\mathrm{dfc}}}^{2}$ is estimated using error propagation from the fitted quantity parameters

(16) $$\sigma_{i_{\mathrm{dfc}}}^{2}=\sum_{i}\sum_{j}\frac{d\delta_{i}}{dQ_{k}}\frac{d\delta_{i}}{dQ_{j}}C_{k,j},$$

(17) $$\frac{d\delta_{i}}{dQ_{k}}=\frac{P(i|k)}{2\sqrt{M_{i}}},$$

where $C_{k,j}$ is the quantity error covariance between *Q_k_* and *Q_j_*.

### 2.7 Significance testing

The $\chi^{2}$ and d.f. given above can be the basis of a $\chi^{2}$ test, with *P*-values being computed via standard spreadsheet functions or look-up tables. The null-hypothesis is always that the fitted LPM is a good description of target data. To test for the significance of particular components, the model can be fitted with and *without* them. With, the $\chi^{2}$ should be consistent with the null-hypothesis, i.e. the resulting distribution of *P*-values should be flat; without, the $\chi^{2}$ should be larger, translating into a small *P*-value. For MS data, components that are prevalent within the areas of pathology can be excluded to form the null-hypothesis of no stroke damage. For the MRI data, the components learned as part of the treatment model can be excluded to form the null-hypothesis of there being no treatment effects.

## 3 Results

Figure 3 illustrates successful estimation of tissue signal quantities and uncertainties using Monte Carlo MS. Visually, there is good correlation between true and estimated quantities—as expected from a pLSA-type analysis. The additional LPM output of an uncertainty image is an honest assessment of the ability to make the measurements. This is corroborated by dividing the difference between the true and estimated images by the uncertainty, giving a pull distribution (Demortier and Lyons, 2002). These should have zero mean (no bias) and their width should be unity (observed spread be equal to those predicted). The pull distributions achieved, being zero-mean, unit-width Gaussians, are evidence that error predictions are indeed correct. The bottom-right pull distribution does show an expected truncation of errors, which is discussed in Section 4.1.

**Fig. 4.**
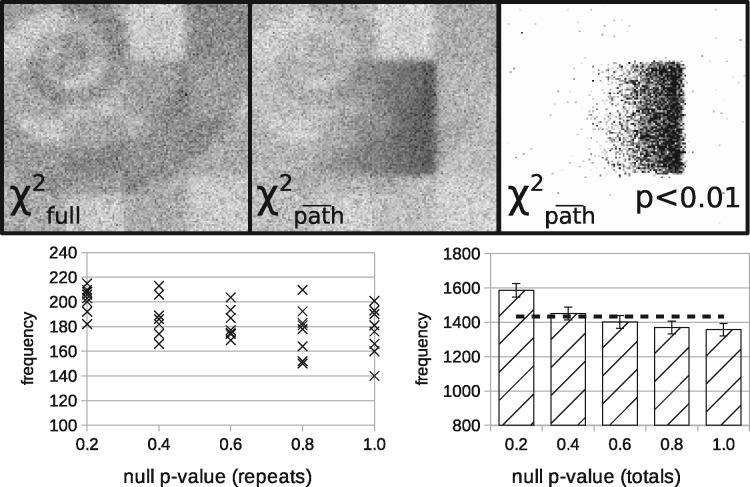
Hypothesis testing in simulation. Top row: $\chi_{\mathrm{full}}^{2}$ shows spatial map of $\chi^{2}$ values when all LPM components are used to describe the data; $\chi_{\bar{\mathrm{path}}}^{2}$ shows the values when the simulated pathology component is removed from the model, giving a null-hypothesis of there being no pathology; *P* < 0.01 shows only values that are significant at the 1% confidence threshold. Bottom row: confirmation that the null-hypothesis test is self-consistent over multiple independent Monte Carlo datasets when the null-hypothesis is true. Dotted line shows expected flat distribution

Figure 4 shows hypothesis testing in simulation. The structure seen within the $\chi_{\mathrm{full}}^{2}$ image reflects the changing d.f. from region to region as the dimensionality of the data changes with the linear composition of the three tissue types. This image acts as a basic goodness-of-fit showing that the LPM is correctly describing the data. If the simulated pathology component is removed, this gives the $\chi_{\bar{\mathrm{path}}}^{2}$ image for the null-hypothesis that there is no pathology. This image shows larger values following the increased quantity of simulated pathology (rectangle with gradient), as the model deviates further from the null-hypothesis. With knowledge of the per-spectrum d.f., the *P* < 0.01 image shows the spectra that reject the null-hypothesis at the 1% confidence level. As a consistency check, the distribution of *P*-values should be uniform when the null-hypothesis holds true.

**Fig. 5.**
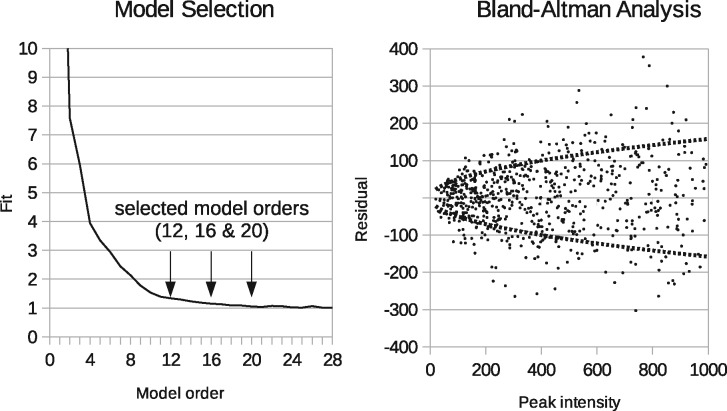
Left: model selection curve showing goodness-of-fit ($\chi_{D}^{2}$) as a function of model-order *N* for rat brain image. Right: Bland–Altman analysis of MALDI MS, as corroborated in earlier work (Deepaisarn *et al.*, 2018)

For MALDI data, Figure 5 shows the goodness-of-fit (global $\chi^{2}$ per d.f.) as a function of model-order. The shape of this curve, monotonically decreasing, is consistent with LPMs improvement to describe data as the number of components grows. The selected orders (arrows at 12, 16 and 20) are inspected to provide insights into how the model components behave as the goodness-of-fit converges and reaches a plateau by *N* = 20. The right of Figure 5 shows a Bland–Altman analysis of a model fit to MALDI data. The *x*-axis shows peak intensities. The *y*-axis shows deviations between LPM values and true peak heights. The dotted curves show a power-law function fitted to these residuals, revealing a shape that is consistent with the Poisson assumption required for the application of our approach, i.e. residuals grow with the square-root of the signal intensity.

**Fig. 6.**
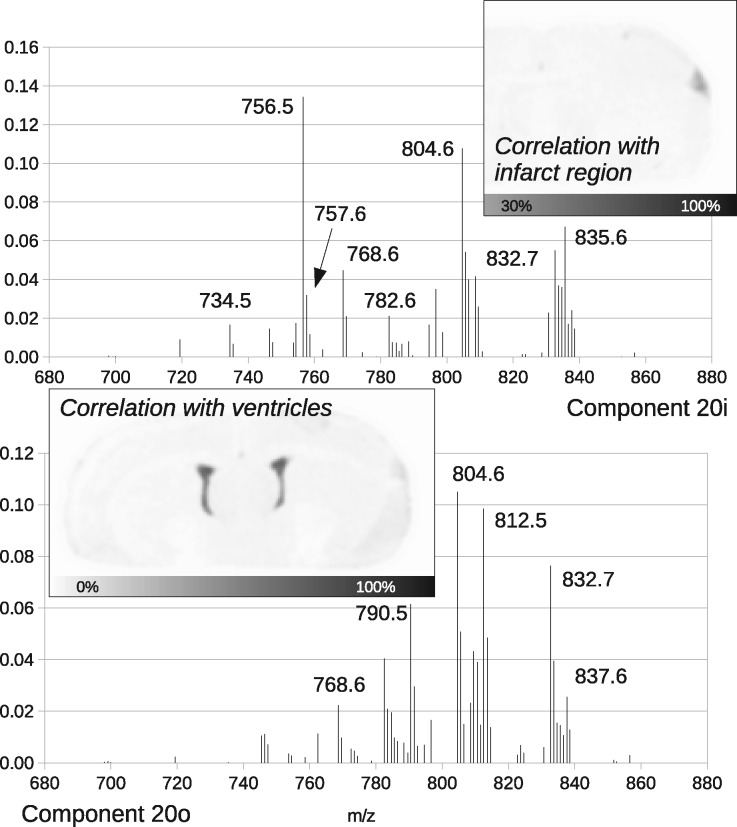
Rat brain components showing highly localized structures and their spectra

A complete set of spectra and images are provided in the Supplementary Material for orders 12, 16 and 20. The grey-scale component images show the relative quantities of each individual component present at each pixel location. The associated spectra are generated by projecting model PMFs onto the original m/z MS axis, showing the relative proportions of each modelled peak. Examples highlighted in Figure 6 show some highly specific correlations as illustrations with ventricles and infarct region. Components are labelled with their model-order and lower-case letter, e.g. 20i is component i from order 20.

**Fig. 7.**
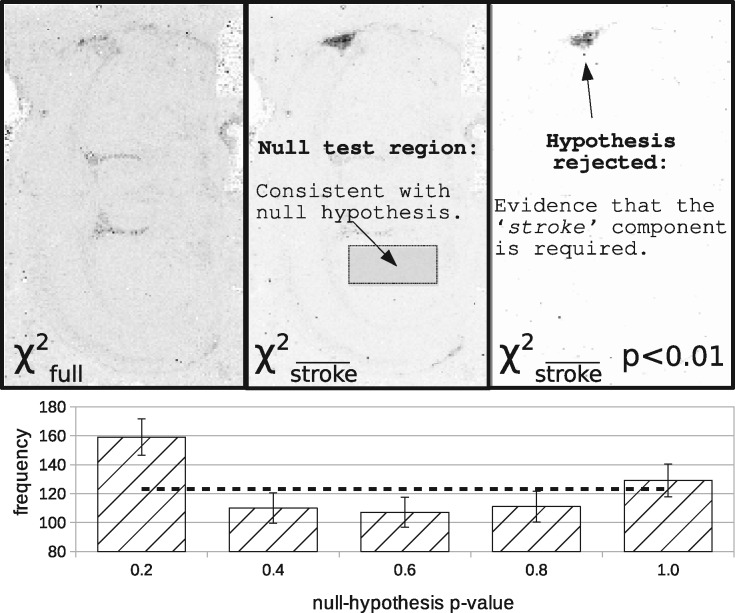
Hypothesis testing in rat brain. Top row: $\chi_{\mathrm{full}}^{2}$ computed from LPM fits including all components. Structure can still be seen within this image as the dimensionality (i.e. d.f.) of the data changes from pixel to pixel. $\chi_{\bar{\mathrm{stroke}}}^{2}$ is computed excluding the ‘stroke’ component, resulting in a null-hypothesis that there is *no* infarct region. *P* < 0.01 shows values only where the null-hypothesis is rejected. Bottom: The distribution of *P*-values within a distal brain region with respect to the known infarct should be flat. The dotted line shows the expected flat distribution

Figure 7 shows the results of hypothesis testing in real data, mirroring the Monte Carlo results shown in Figure 4. As this is real data, there is no control over the true proportions of tissue types, but it is assumed that there is zero pathology away from the infarct region. To test the *P*-value distribution under the null-hypothesis a region distal to the infarct is therefore selected, which is uniform.

**Fig. 8.**
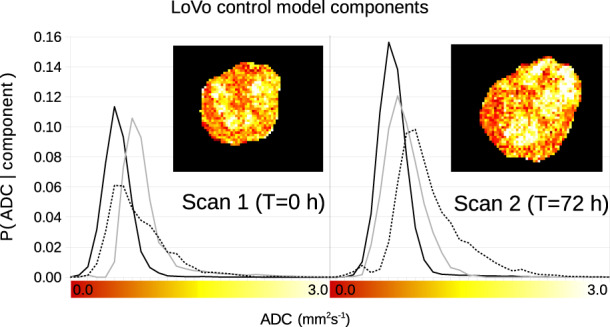
Example LPM components of the ADC distribution for LoVo control tumours

Figure 8 illustrates the type of LPM components generated for the second dataset describing the voxel-wise ADC distributions of tumours. The example given shows three modes of correlated variation found within the LoVo control cohort of tumours, along with example slices of a specific tumour at baseline and 72 h. Low ADC values show low mobility of water, consistent with dense tumour tissue. Higher values reflect more free diffusion, e.g. in necrotic tumour. In this supplementary dataset, an ADC distribution takes on the role previously played by an MS image pixel, but note that there is only one ADC distribution per tumour—we are not modelling individual pixels in this case.

**Fig. 9.**
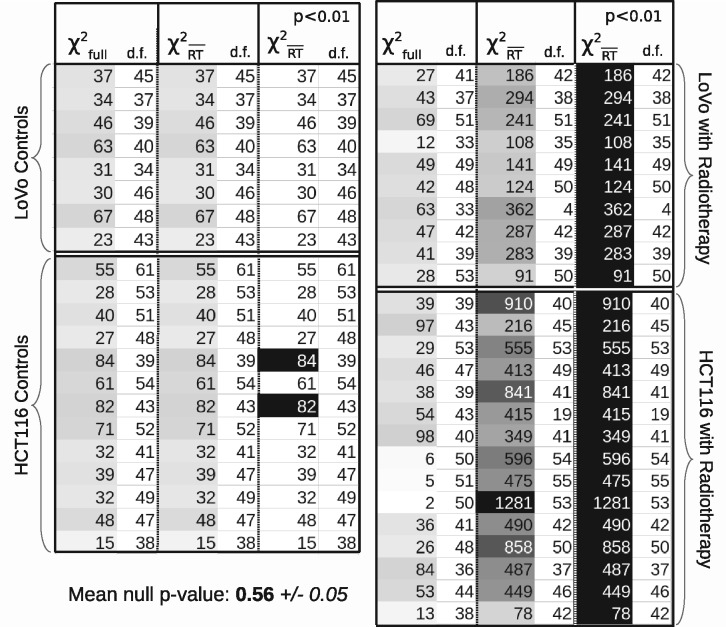
Control and treated tumour cohort distributions of $\chi^{2}$ values and d.f. estimates. $\chi_{\mathrm{full}}^{2}$ are values from residuals using a complete LPM model, containing both control behaviour and treatment behaviour components. $\chi_{\bar{RT}}^{2}$ are values using only control behaviour, i.e. no RT. *P* < 0.01 thresholded values shown with black background

Figure 9 shows the results of $\chi^{2}$ testing applied to control and treated tumours, under the null-hypothesis that no treatment effects are present. The figure follows the convention set by Figures 4 and 7, with $\chi^{2}$ of residuals computed using a full LPM model, a smaller model excluding components, in this case excluding the treatment-related components, and finally a grey shading level, turning black for those passing the 1% significance level. The left-hand set of ‘control’ results reveals two possible outliers in training data. However, the mean *P*-value over all controls is consistent with a half (which is consistent with a flat hypothesis distribution). All treated tumours reach the 1% significance level, as expected.

## 4 Discussion

LPM adds important functionality to pLSA making it a more powerful and useful tool for describing MS images. The addition of quantity error estimates and the ability to conduct hypothesis testing facilitates a more scientific approach to the quantification of signals within biological MS, as demonstrated in this study. This is made possible through an improved understanding and modelling of uncertainties, and the reduction of linear degeneracies through the use of MAX SEP. We believe that the benefits and potential uses for this new technique will enable novel future applications as discussed below. Evidence from MRI, as a second dataset further suggests potential for more generic histogram analysis.

### 4.1 Use of quantity error estimates

LPM provides a set of uncertainty images. These images, one per linear component, provide a spectrum-by-spectrum account of the errors expected on pixels’ associated quantity/proportion estimates. A clear example of this can be seen in Figure 3. In this Monte Carlo example, it is not sufficient to assume a fixed error, or even Poisson errors on estimated quantities. The map of uncertainties changes as a function of quantity and level of ambiguity with other components. This is visible as a ghostly superimposition of other components, perhaps most clearly seen in the central spiral uncertainty map, where a slight chequer pattern can also be seen. The size of the errors and correlations between them is described by a full error covariance matrix that can be used to either simply place error bars on measurements, or to assist in further analysis.

The pull distributions in Monte Carlo show that the error images give a true reflection of the uncertainties found within estimated quantities. However, the simulated pathology component (central rectangle with a quantity gradient from left to right) shows a pull distribution that is truncated. This is an expected effect, as the true errors in quantities cannot go negative due to the positive-only nature of spectra. This has the effect of making error estimates close to zero quantities positively biased. Despite this, it can be seen that the pull distribution is otherwise correctly shape, bar the missing tail on the right.

Noise correlations in data (or similarly double counting) effectively change the number of unique data points (or d.f.) in an image. LPM assumes each spectra peak is an independent variable. Assuming any correlation is on average fixed across an image (either between peaks or between pixels) scaling by the $\chi^{2}$ per d.f. will compensate. Furthermore, residual correlations caused by parameter fitting are dealt with directly through our per-spectrum d.f. estimation.

### 4.2 Use of hypothesis testing

One may consider thresholding a quantity image as a surrogate for a statistical hypothesis test. However, the varying errors from spectrum-to-spectrum would result in inconsistent confidence levels. One may consider thresholding the $\chi^{2}$ images, but this too would not result in a stable test because of d.f. changes. The method presented here, however, gives meaningful *P*-values by using $\chi^{2}$ and correct d.f. estimates.

For ‘pathology’ hypothesis testing, a component needs selecting. 20i was selected as it correlates well with the known location of induced stroke damage. It also does not appear in any significant quantities beyond the stroke region. In general, the test only determines the significance of the signal associated with the chosen part of the model, but can say nothing regarding the biological nature of the component. To be sure that a component represents pathology of interest further investigation is likely needed, such as histology. The 20i is a sufficient component to test *P*-value calculations, but users in practice are responsible for making a positive identification of links with biology. Removing 20i generated the null-hypothesis that there was no stroke damage.

The 99th percentile confidence threshold applied to the rat brain clearly highlights the stroke-damaged region, showing that the component 20i was required (rejection of null-hypothesis). In addition, it shows a scattering of high-confidence pixels in other parts of the image. With 25 000 spectra, it would be expected that around 250 spectra would pass the threshold due to noise alone, so these are to be expected. Furthermore, hypothesis testing on our ADC tumour data reveals high levels of significance for every treated tumour, in both the LoVo and HCT116 models, whilst showing correspondingly low significance in controls. A user of the method must be aware that making use of multiple *P*-values may require corrections for multiple-comparisons. We consider this to be an application-specific problem to be considered on a case-by-case basis.

The most compelling quality control test used is the creation of hypothesis probability distributions, which under the null-hypothesis should be uniform. In both Monte Carlo and in the rat brain data, areas of the image believed to be consistent with the null-hypotheses were indeed found to be flat. This would not be the case if any of the LPM statistical assumptions were violated.

### 4.3 Model validation

Like all non-trivial analyses, using LPM requires quality control to ensure results are trustworthy. Steps must be taken to ensure statistical assumptions are met, as failure to do so can invalidate conclusions. Possible problems can include: non-Poisson variations; insufficient model fits caused by local minima; correlations within source data; and correlations between residuals, as discussed below.

There is risk that non-Poisson variations dominant uncertainty in some MS data due to variations in sample preparation and pre-processing methods. To mitigate this, certain variations can be modelled as additional linear components, such as the potential chemical noise found in components 12a, 16a and 20p in the Supplementary Material. Carefully applied pre-processing, specifically designed to maintain desire statistical properties, also helps. Bland–Altman analysis shows that our pre-processed MALDI spectra exhibits Poisson sampling behaviour.

Insufficient fits occur if the model-order is too low or local minima are found. Checking for this is confounded by scaling of data where a unit of signal is not always equivalent to an ion count, e.g. 1 count is typically a step-change in voltage, not a simple counter increment. For error predictions and hypothesis testing to work correctly, scaling of this type must be known. Using a $\chi^{2}$ per d.f. for model selection allows both the model-order and possible scaling to be determined together, as the plateau to the right of the model selection curve can be used as a scaling factor. The 20 component model satisfied all of our quality control measures and thus was deemed safe to use. A poor model would have resulted in poor quantity error predictions, and thus poor d.f. estimates, leading ultimately to a non-uniform distribution of *P*-values.

### 4.4 Scalability and performance

The LPM method has the same time-complexity as pLSA and NNMF. To build a single model, run-time grows linearly with the number of mass bins, number of spectra and model-order. The slowest part of the modelling process is determining model-order, as a range of orders must be tested. Avoiding local minima is achieved by building multiple models and selecting the one with the lowest $\chi^{2}$, which can be a slow process. To ensure a good solution was found, 50 models were built per model-order. Using up to 4 machines (Dell Precision T7500s), this process took up to a week for model-orders containing 12+ components. However, the much smaller tumour data required only minutes, due to the small number of tumours typically found in pre-clinical trials, and also the modelling of per-tumour, rather than per-pixel ADC distributions.

Focusing on a relatively small number of major peaks is a strategy for speeding up model building. We suggest that if a large number of minor peaks are important for answering certain biological questions then a piece-meal approach can be taken. Multiple models can be constructed to inspect different areas of the data, with the hypothesis testing parts of the process remaining the same for any desired null-hypothesis component.

## 5 Conclusion

As bio-images become ever larger and more complex, researchers are increasingly turning to pattern recognition tools for dimensional reduction and for extracting summary measurements. Advanced approaches, including pLSA and deep learning, may extract invariant characteristics (e.g. for identifying tissue-specific MS), and may also provide indicative labels for categorizing local data points (e.g. pathological tissues). However, as we have shown, there is an additional layer of complexity that must be addressed if outputs are to satisfy scientific requirements; in particular, the requirement of providing *P*-values to quantify the significance of results.

We have shown that through careful modelling of signal, and control of statistical characteristics, we can construct valid hypothesis tests for different bio-images. Our method of $\chi^{2}$ testing for pLSA-type descriptions of data has been validated on single parameter MRI scans, and also large hyper-spectral MS images. In the former, heterogeneous changes in tumours over time allowed treatment effects to be detected with high significance (*P* < 0.01), despite the limited training data and the chaotic nature of tumours. In the latter, a data-rich MALDI brain image, with its regular anatomy and well-defined tissues, illustrated our ability to spatially map pathology with high significance. These very different images suggest LPM analyses could have wide ranging applicability—albeit with appropriate quality controls.

## Supplementary Material

btaa270_Supplementary_Data
