# Supplementary material for: A reformulation of pLSA for uncertainty estimation and hypothesis testing in bio-imaging
Source: Bioinformatics. 2020 Apr 23;36(13):4080–7. doi: 10.1093/bioinformatics/btaa270 (PMC7332574; doi:10.1093/bioinformatics/btaa270)
Supplement: btaa270_Supplementary_Data [file btaa270_supplementary_data.pdf]

Bioinformatics
doi.10.1093/bioinformatics/xxxxxx
OXFORD

ADDITIONAL

A reformulation of pLSA for bio-imaging, uncertainty estimation and hypothesis testing

P.D. Tar*, N.A. Thacker, S. Deepaisarn, J.P.B. O'Connor, and A.W. McMahon

Division of Informatics, Imaging and Data Sciences, The University of Manchester, Manchester, UK
Division of Cancer, The University of Manchester, Manchester, UK

Abstract

Motivation: Probabilistic Latent Semantic Analysis (pLSA) is commonly applied to describe mass spectra (MS) images. However, the method does not provide certain outputs necessary for the quantitative scientific interpretation of data. In particular, it lacks assessment of statistical uncertainty and the ability to perform hypothesis testing. We show how Linear Poisson Modelling (LPM) advances pLSA, giving covariances on model parameters and supporting χ^2 testing for the presence / absence of MS signal components. As an example, this is useful for the identification of pathology in MALDI biological samples. We also show potential wider applicability, beyond mass spectra, using MRI colorectal tumor data.
Results: Simulations and MALDI spectra of a stroke-damaged rat brain show MS signals from pathological tissue can be quantified. MRI diffusion data of control and Radiotherapy-treated tumors further show high sensitivity hypothesis testing for treatment effects. Successful χ^2 and degrees-of-freedom are computed, allowing null hypothesis thresholding at high levels of confidence.
Availability: Open source image analysis software available from TINA Vision, www.tina-vision.net
Contact: paul.tar@manchester.ac.uk
Supplementary information: Supplementary material is available at *Bioinformatics* online.

1 Spectra and images

The following images and spectra present results from LPM model orders 12, 16 and 20. Each figure contains the spectrum of the component and an image showing its relative abundance. Component labels start with the model order and are followed by the component's letter, e.g. '12h' is component h from model order 12. The most significant peaks within each spectrum are additionally labelled with their m/z value. The image intensity is inversely proportional to its abundance, with white representing 0% of the component appearing at a location and black representing 100%.

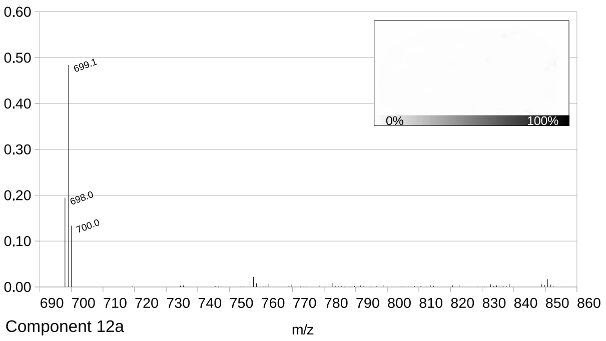

Fig. 1.

© The Author 2019. Published by Oxford University Press. All rights reserved. For permissions, please e-mail: journals.permissions@oup.com

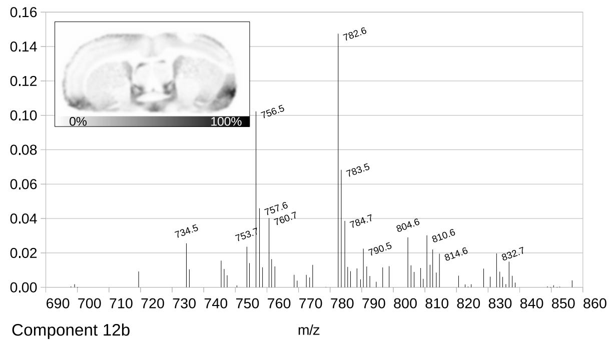

Fig. 2.

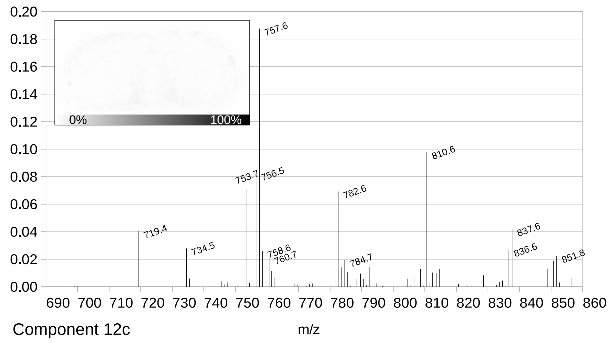

Fig. 3.

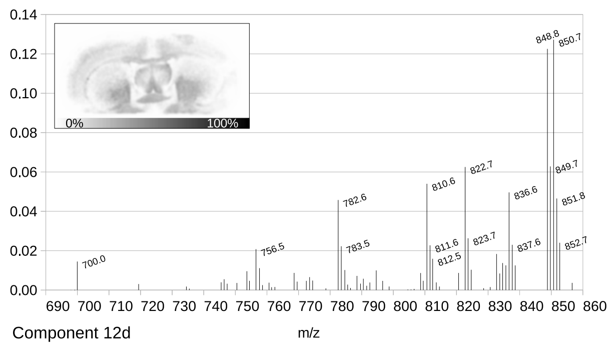

Fig. 4.

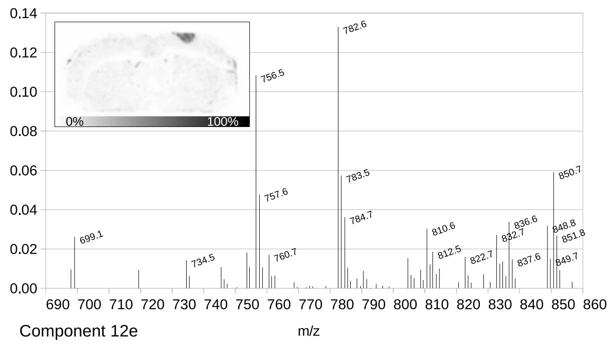

Fig. 5.

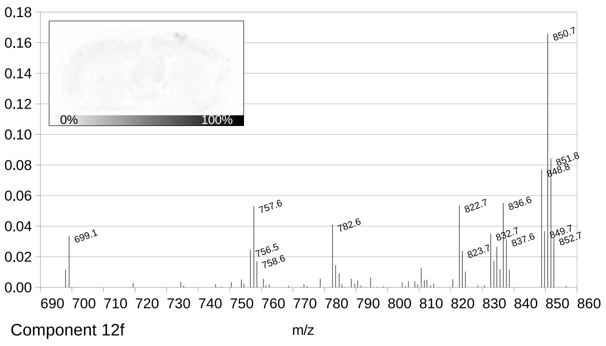

Fig. 6.

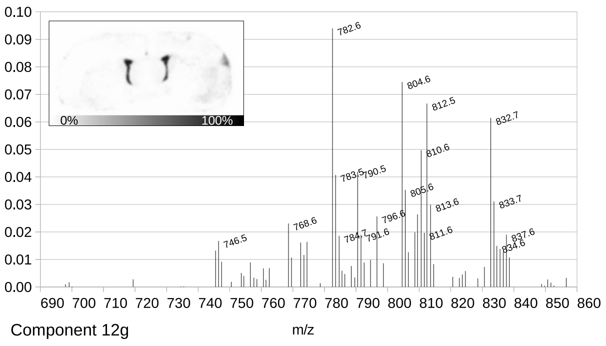

Fig. 7.

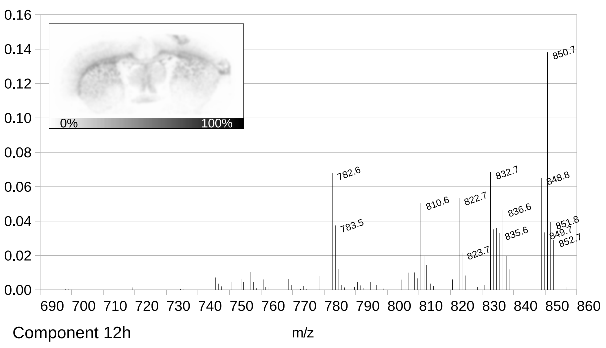

Fig. 8.

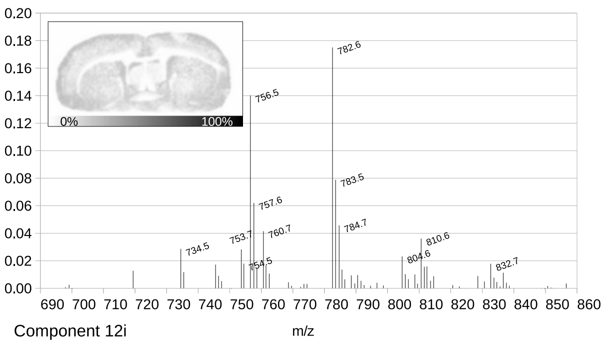

Fig. 9.

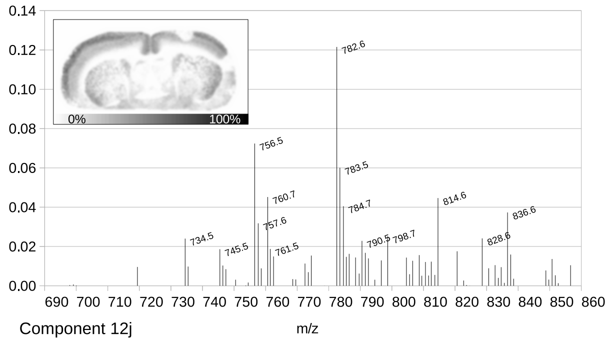

Fig. 10.

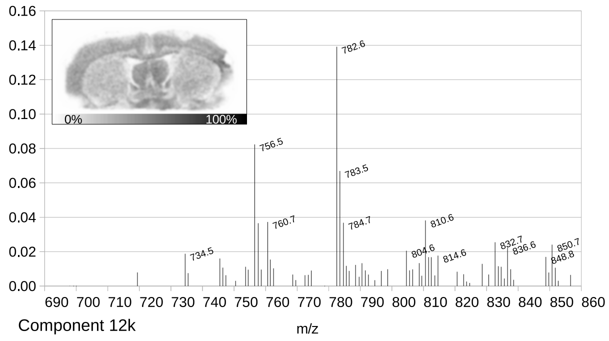

Fig. 11.

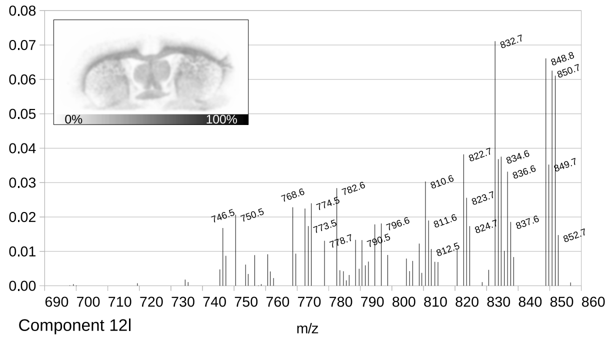

Fig. 12.

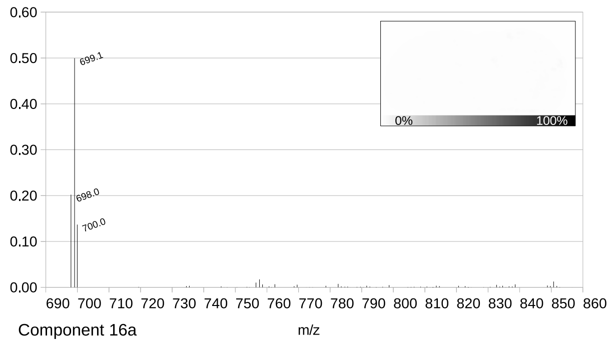

Fig. 13.

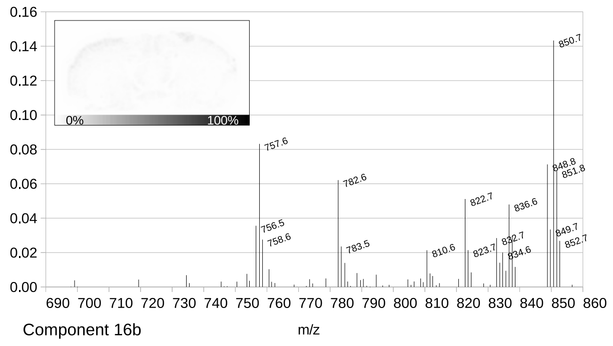

Fig. 14.

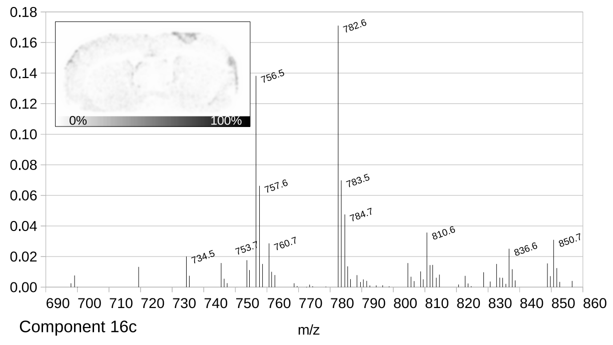

Fig. 15.

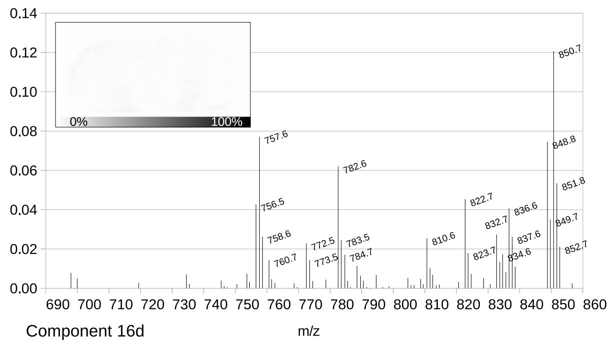

Fig. 16.

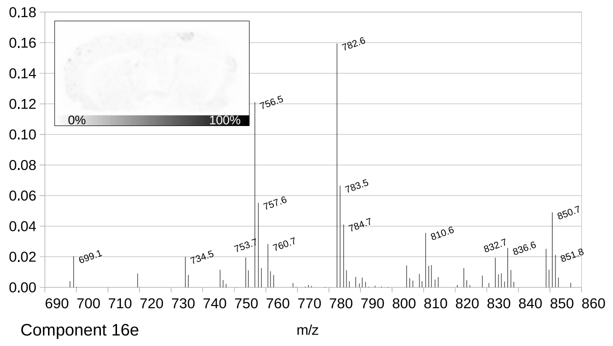

Fig. 17.

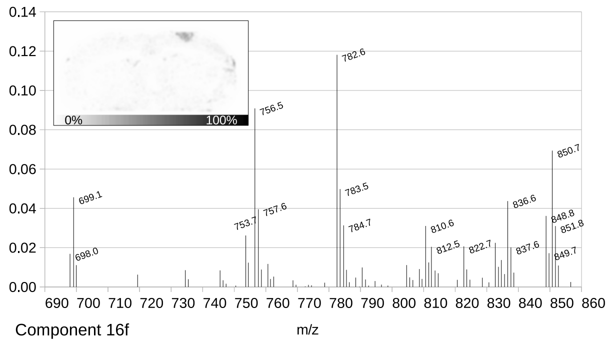

Fig. 18.

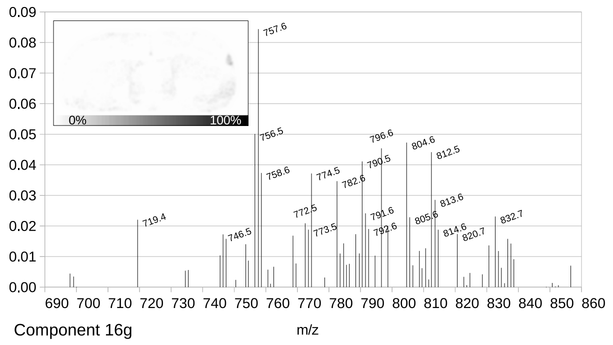

Fig. 19.

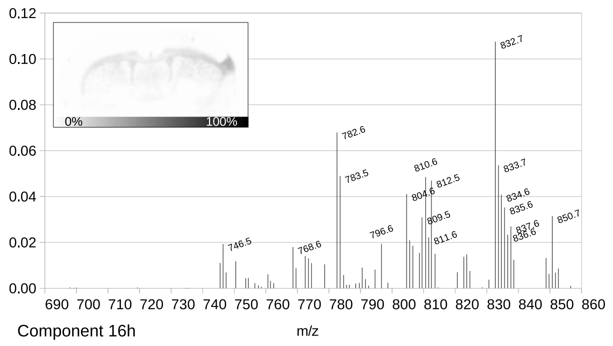

Fig. 20.

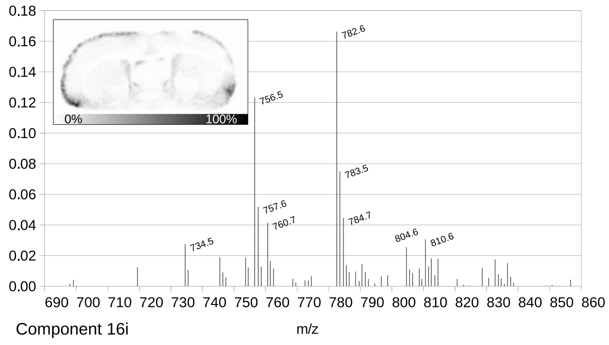

Fig. 21.

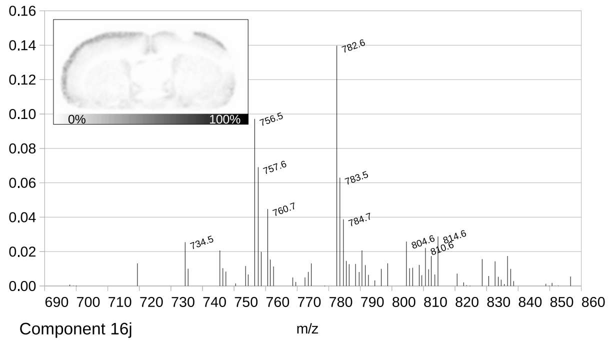

Fig. 22.

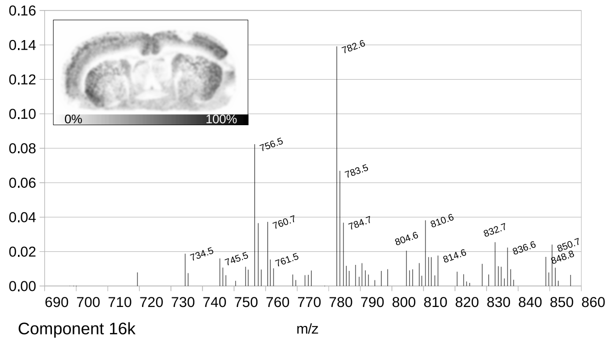

Fig. 23.

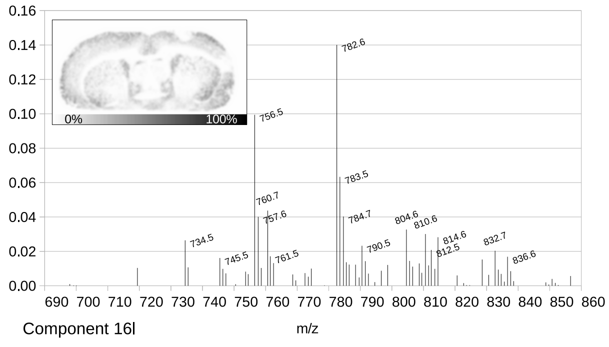

Fig. 24.

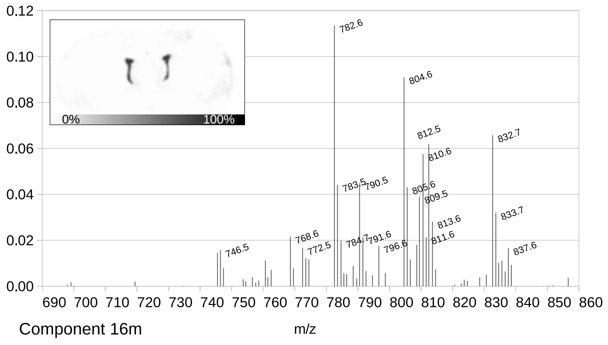

Fig. 25.

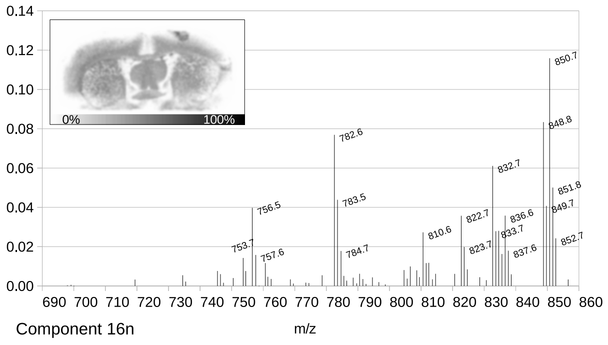

Fig. 26.

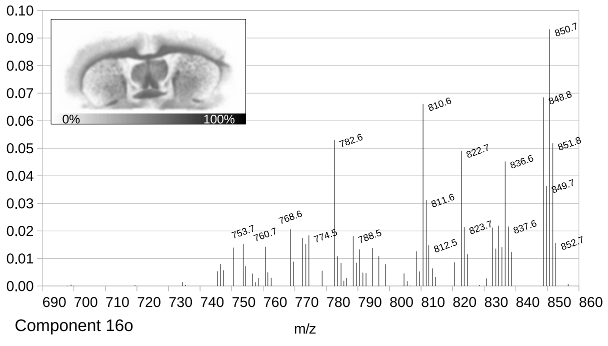

Fig. 27.

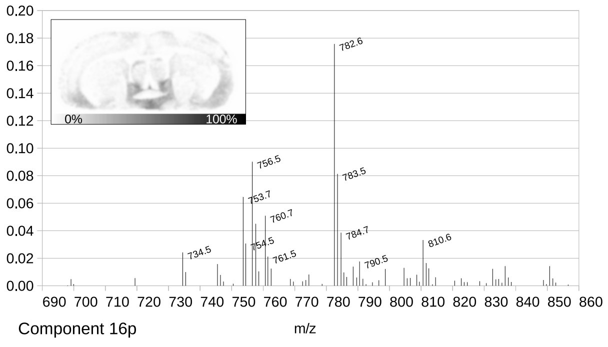

Fig. 28.

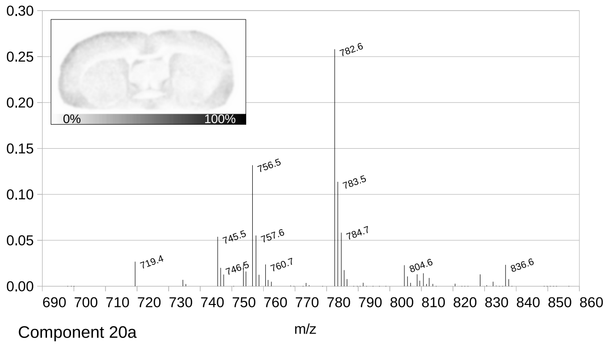

Fig. 29.

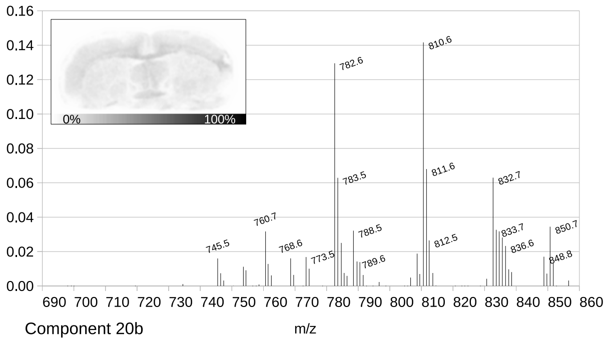

Fig. 30.

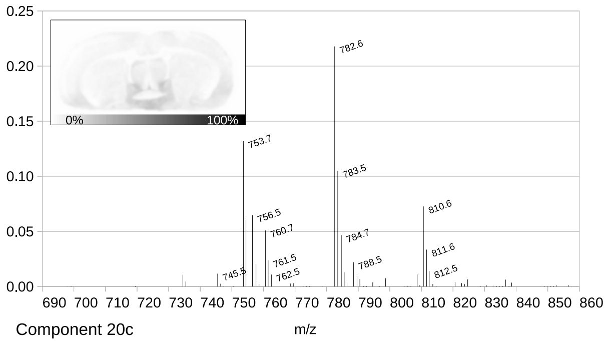

Fig. 31.

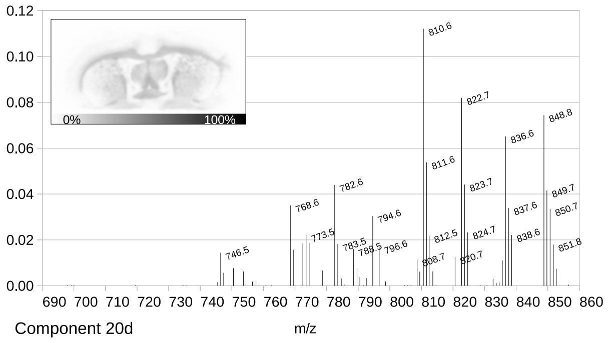

Fig. 32.

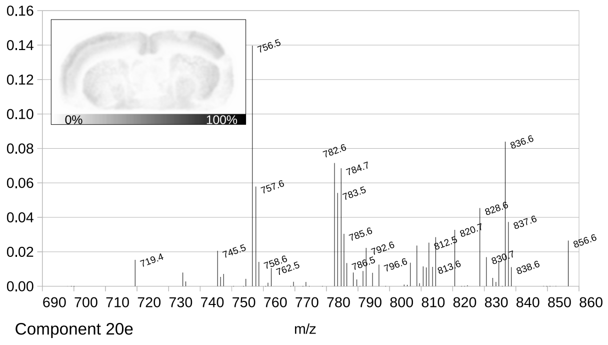

Fig. 33.

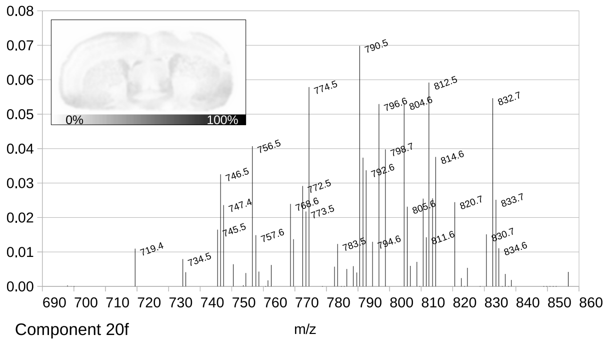

Fig. 34.

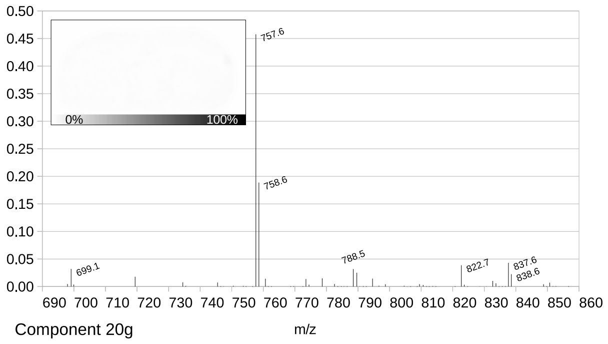

Fig. 35.

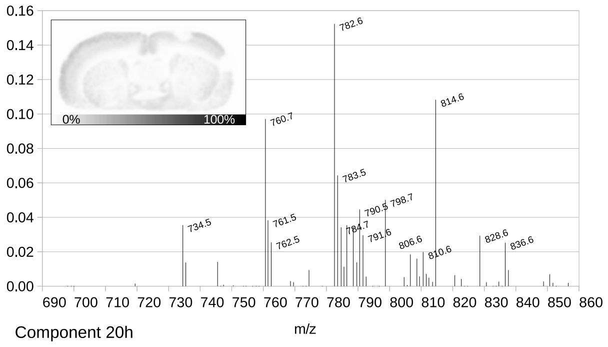

Fig. 36.

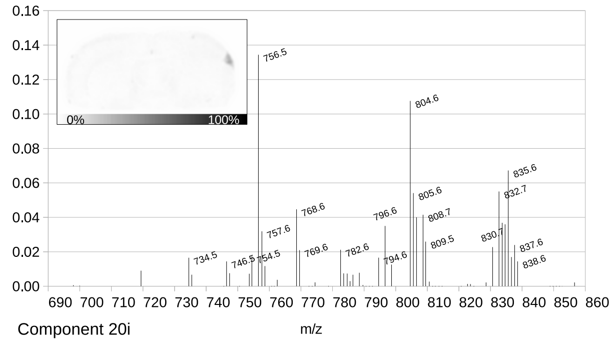

Fig. 37.

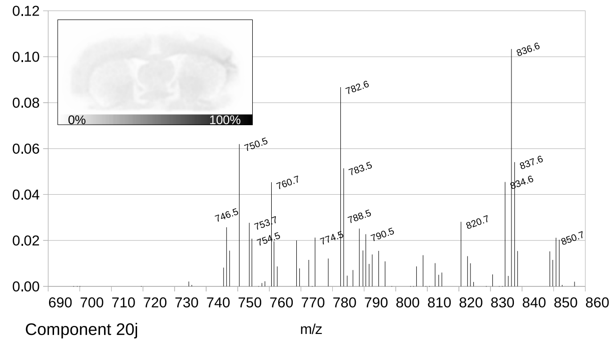

Fig. 38.

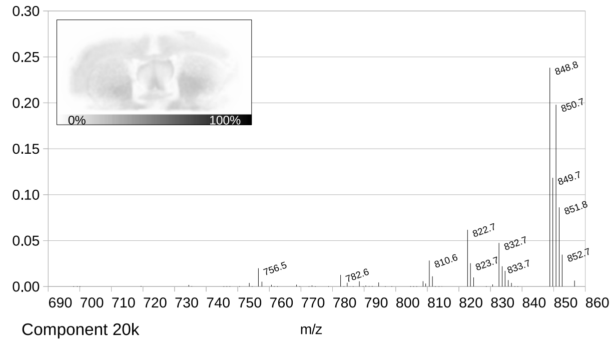

Fig. 39.

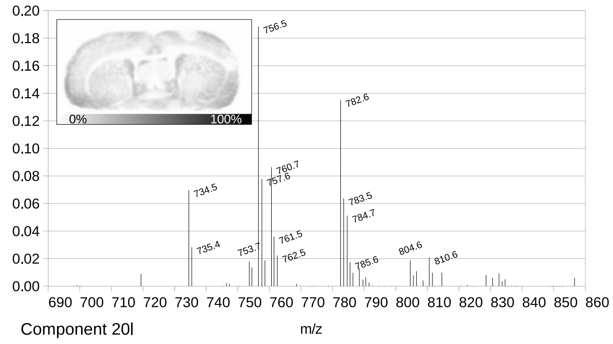

Fig. 40.

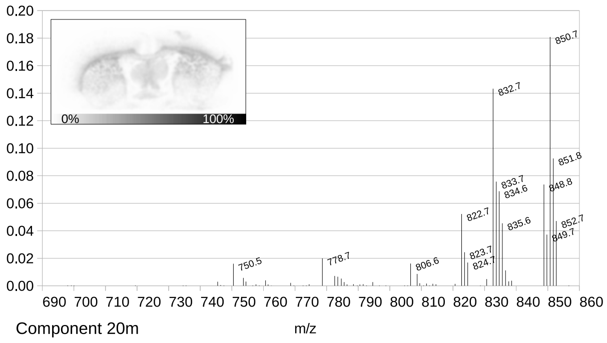

Fig. 41.

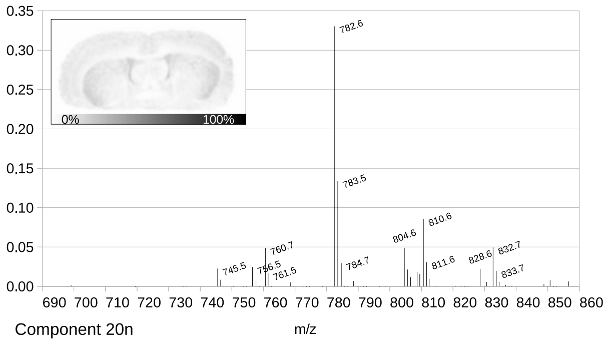

Fig. 42.

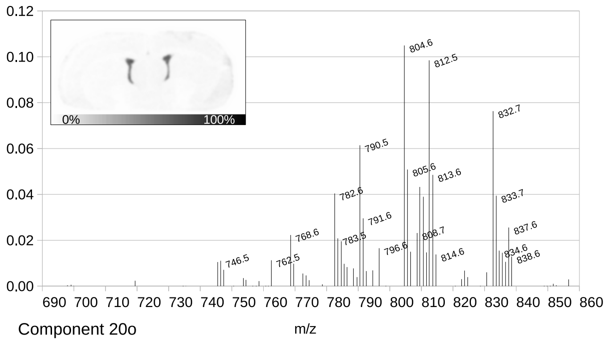

Fig. 43.

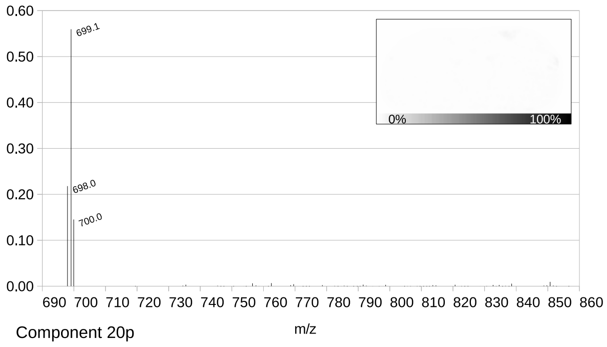

Fig. 44.

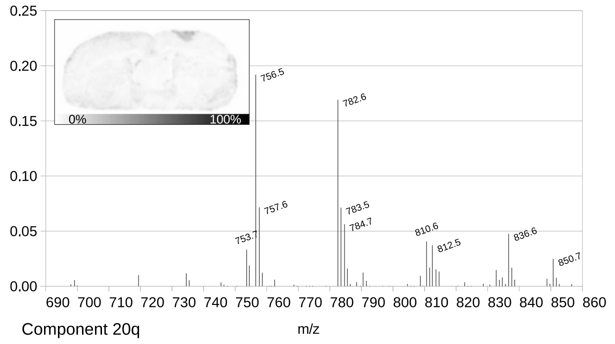

Fig. 45.

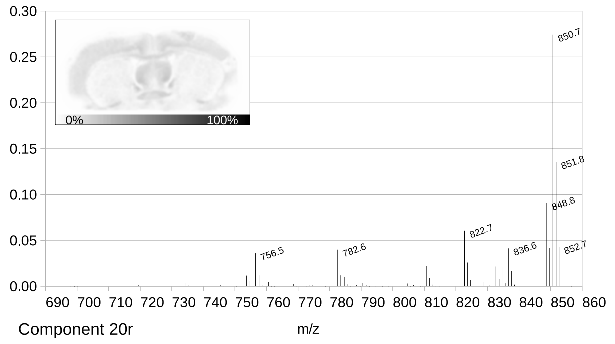

Fig. 46.

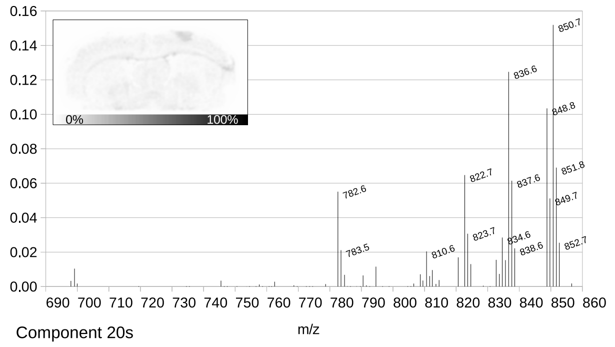

Fig. 47.

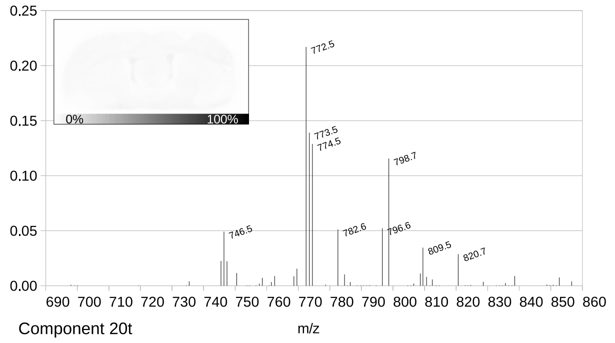

Fig. 48.

2 Tumor ADC histograms and models

To demonstrate the wider potential of the hypothesis testing method, we present a non-mass spectra dataset. Our secondary dataset replaces the mass spectra histograms with sampled distributions from pre-clinical MRI scans. We use a mode of imaging known as Apparent Diffusion Coefficient (ADC), which is commonly applied in cancer studies. High ADC values correspond to areas of tumors with water molecules that are more mobile. Low values correspond to dense areas with less fluid motion. As tumor tissue dies and become necrotic, it tends to become less dense and more fluid. This makes ADC a useful biomarker for the study of treatment effects. In our demonstration, we use changes in ADC over time to show how LPM can produce a sensitive hypothesis test for treatment response, under the null-hypotheses that there are no treatment effects between two scanning sessions.

Colorectal tumors, implanted into mice (LoVo and HCT116), were divided into control and treatment groups. The treatment groups were given radiotherapy, whereas the control groups received no treatment. Each tumor was scanned at baseline (pre-treatment) and 72 hours later. Scan pairs were used to produce histograms suitable for use with LPM. Whereas LPM models of spectra attempt to extract physically meaningful correlations between detected ions and their abundances in different tissues, in contrast, the ADC models extract correlated changes between scanning visits associated with control behaviour and treatment response. To achieve this, the data from scan pairs are sampled into 2D histograms, with ADC on one axis and visit number on the other. Each tumor thus produces a histogram, which then plays a similar role to what previously was a mass spectrum image pixel.

The LPM models were constructed in two parts: Firstly, histograms of control tumors only were input. This provided a set of LPM components associated with control growth. It was anticipated that treated tumors would exhibit some form of control development, along with additional modes of variation specific to treatment. Secondly, to model this, control components were introduced as immutable parts of a wider 'control plus treatment' model. The treated histograms were input and further components, in addition to the predetermined control components, were extracts to describe the additional variations.

The resultant components are fitted to control cohort and treatment cohort histograms in various combinations to construct different hypothesis tests using a 'control-only' and a 'control-plus-treatment' model. Note that the null-hypotheses is always that the components fitted are the only ones needed to describe the data, thus:

- when the control-only model is fitted to control data, the null-hypotheses should be satisfied, as there is, by definition, no treatment effects;
- when the control-plus-treatment model is fitted to the treated data, the null-hypotheses should also be satisfied, as both control and treatment-related variations are accounted for;
- however, when the control-only model is fitted to the treated data, the null-hypotheses should be rejected, as this results in larger than expected χ^2 values, and thus more significant p-values.

These combinations can be seen in the main paper, showing significant results for treatments, and evidence of a uniform hypothesis distribution when the null-hypotheses holds (i.e. mean null p-value consistent with a half).

These results are extensions of those provided in Tar et al. 2018 'A new method for the high-precision assessment of tumor changes in response to treatment', bioinfo. In this former work, volumetric estimates of tumor response were provided and a full LPM model was required. Here, only a control model is required to perform hypothesis testing (i.e. corresponding to the third bullet point above). As there is no strict need to build a treatment model in this case, this can simpify the process of testing individual tumors for treatment effects.
